# Identification of skeletal remains in Croatia and Bosnia and Herzegovina, including the homeland war – a 30-year review

**DOI:** 10.3325/cmj.2024.65.239

**Published:** 2024-06

**Authors:** Dragan Primorac, Šimun Anđelinović, Marija Definis-Gojanović, Vedrana Škaro, Petar Projić, Miran Čoklo, Adna Ašić, Bruce Budowle, Henry Lee, Mitchell M Holland, Michael Baden, Damir Marjanović

**Affiliations:** 1 St. Catherine Specialty Hospital, Zabok/Zagreb, Croatia; 2 Eberly College of Science, The Pennsylvania State University, State College, PA, USA; 3 Medical School, University of Split, Split, Croatia; 4 Medical School, Josip Juraj Strossmayer University of Osijek, Osijek, Croatia; 5 The Henry C. Lee College of Criminal Justice and Forensic Sciences, University of New Haven, New Haven, CT, USA; 6 Regiomed Kliniken, Coburg, Germany; 7 Medical School, University of Rijeka, Rijeka, Croatia; 8 Faculty of Dental Medicine and Health, Josip Juraj Strossmayer University of Osijek, Osijek, Croatia; 9 Medical School, University of Mostar, Mostar, Bosnia and Herzegovina; 10 National Forensic Sciences University, Gandhinagar, India; 11 University Hospital Center Split, Split, Croatia; 12 Greyledge Europe Ltd, Zagreb, Croatia; 13 International Center for Applied Biological Sciences Ltd (ICABS), Zagreb, Croatia; 14 Institute for Anthropological Research, Zagreb, Croatia; 15 Verlab Research Institute, Sarajevo, Bosnia and Herzegovina; 16 University of Helsinki, Department of Forensic Medicine, Helsinki, Finland; 17 Henry C. Lee College of Criminal Justice and Forensic Sciences, University of New Haven, West Haven, CT, USA; 18 Forensic Science Program, Biochemistry & Molecular Biology Penn State University, State College, PA, USA; 19 New York State Police Forensics Division, New York, NY, USA; 20 Department of Genetics and Bioengineering, International Burch University, Sarajevo, Bosnia and Herzegovina, Croatia; 21 Faculty of Biotechnology and Drug Development, University of Rijeka, Rijeka, Croatia

## Abstract

Over the past 30 years, forensic experts from Croatia and Bosnia and Herzegovina have embraced advanced technologies and innovations to enable great efficacy and proficiency in the identification of war victims. The wartime events in the countries of former Yugoslavia greatly influenced the application of the selected DNA analyses as routine tools for the identification of skeletal remains, especially those from mass graves. Initially, the work was challenging because of the magnitude of the events, technical aspects, and political aspects. Collaboration with reputable foreign forensic experts helped tremendously in the efforts to start applying DNA analysis routinely and with increasing success. In this article, we reviewed the most significant achievements related to the application of DNA analysis in identifying skeletal remains in situations where standard identification methods were insufficient.

Almost 30 years have passed since the initial identification of the skeletal remains of war victims from the former Yugoslavia region. In these three decades, scientists from Croatia and Bosnia and Herzegovina (B&H) cooperated with the leading forensic scientists, primarily from the US, to achieve what many thought would be impossible. At the end of the 20th century, DNA analysis was already well-established as a promising new method for human identification and forensic individualization. Still, the Armed Forces DNA Identification Laboratory in the US was the first institution to begin using nuclear and mitochondrial DNA analysis to identify human skeletal remains (1,2).

Following the initial hurdles and technical obstacles, scientists from Croatia and B&H succeeded in their mission – to identify the victims and thus allow them a dignified burial. Their efforts also helped alleviate the local community's anguish, particularly for those seeking closure regarding the fate of their family members. The first successful DNA identification of large-scale mass grave victims (skeletal remains) was performed in the laboratory of the Department of Pathology and Forensic Medicine, University Hospital Split, Split, Croatia. Three persons found in a Kupres mass grave (B&H) were identified (3,4). Soon after, the use of STR DNA typing system to identify mass disaster victims entered the forensic field. This began a new era for the Laboratory of Clinical and Forensic Genetics, significantly affecting the advancement of clinical sciences and forensics, both in scientific and educational terms, as well as in a professional capacity (5). The scientific advancements stemming from these missions were subsequently applied globally, from the identification of the victims of terrorist attacks in New York City and Washington, D.C. (6) to those of tsunamis and wars in Iraq, Syria, and Libya. This also extended to the initial identifications of civilian victims from mass graves dating back to the Second World War in Slovenia, Croatia, and B&H.

Here, we compiled some of the most interesting articles from that period, so that this review article may serve as a single source of information about these pioneering efforts for current and future scientists. We did not manage to collect all the scientific articles, nor were we able to mention all the scientists who contributed to DNA identification of war victims in this region. However, let this paper serve as an initial effort in documenting a successful international scientific cooperation that offered the local communities much more than scientific results.

## PRE-DNA EFFORTS

The dissolution of Yugoslavia at the end of the 20th century triggered a range of war activities, which were centralized mainly in Croatia and B&H between 1991 and 1995, as well as in Kosovo in 1999. These wars resulted in over 200 000 deaths (7) and an estimated 40 000 missing individuals (8). During the Homeland War in Croatia, more than 20 000 persons lost their lives (9). Due to the efforts of the relevant authorities of the Republic of Croatia, the fate of most of the registered missing persons has been resolved. However, 1418 persons are still missing, and the burial place of 394 deceased persons remains unknown, which adds to 1812 unsolved cases from the Croatian Homeland War (10). During the war in B&H, close to 100 000 individuals lost their lives (11). Around 80% of all missing individuals have been successfully identified, but almost 7600 people are still missing (12).

Identification of war victims presents a great challenge for many reasons, especially since the remains of tens or, even hundreds, of individuals may be found within a single mass grave. Therefore, multiple methods were used in the identification efforts (3,13-16). These methodologies encompass various techniques, such as dental analyses, fingerprinting, and direct facial recognition by a living person. They also involve examining skeletal remains by forensic anthropologists (including estimation of age, biological sex, time of death, stature, and population affinity), autopsy, reconstruction of facial features from the skull, identifying personal traits like tattoos and scars, comparing hair samples, recognition of personal effects such as clothing and attire through witness testimony, and DNA analysis. The selection of an appropriate method and its effectiveness depend on the circumstances and condition of the remains being examined. These methods and challenges were discussed in the first publications on the identification efforts in Croatia and B&H (15,17).

Strinović et al reported on the first identification in pre-DNA time in 1994. It was performed at the Institute of Forensic Medicine and Criminology in Zagreb, Croatia, resulting in 73/110 successful identifications. Some bodies were delivered in groups, while others were delivered individually. The largest group consisted of 20 members of the Croatian army killed in the village of Kusonje; they were delivered five months following their deaths (17). The researchers compared fingerprints, dental records, special body features and items (tattoos, jewelry), available documents, and visual recognition (17). However, none of these methods was completely suitable, and most had significant drawbacks when applied to war victim identification.

**Figure 1:**
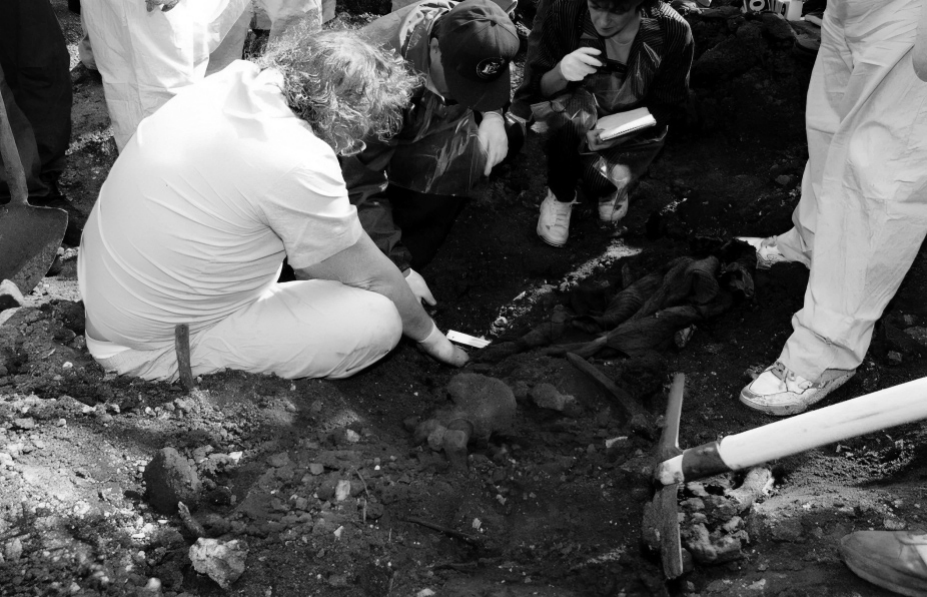
Excavation from Kupres (Bosnia and Herzegovina) mass grave, 1993.

**Figure 2:**
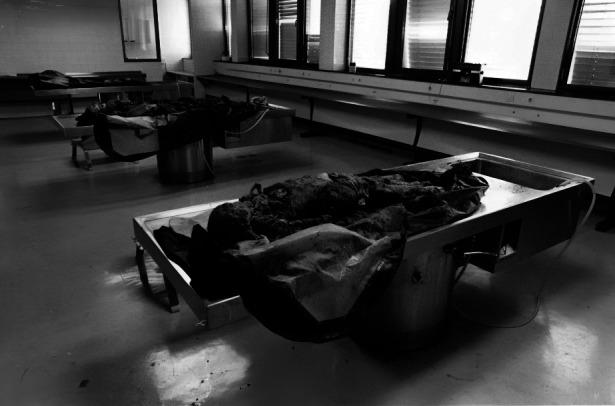
Skeletal remains from Kupres (Bosnia and Herzegovina) mass grave at the Department of Pathology and Forensic Medicine, Clinical Hospital Center Split, Split, Croatia, 1993.

Another group of scientists reported on the identification of the very first casualties of the war in Bosnia and Herzegovina in 1995 (15). They examined 59 victims from the mass graves located in the Kupres area, West Bosnia (Figures 1 and 2).

The identification methods employed were similar to those described above, including recognizing personal items (clothes, footwear, jewelry, documents), stature and hair analysis, body marks, dental records, x-ray analysis, and video superimposition (Table 1). Although as a result of such a multidimensional approach, 35 persons were positively identified, 24 remained unidentified (15).

**Table 1:** Methods used in complex pre-DNA identification in Croatia. Identification methods of war victims from the Kupres region (15). Positive identification included at least two methods

Method	Number of identified persons
Stature	32
Clothes/footwear	30
Jewelry and other personal belongings	16
Dental status	14
Hair	8
Special marks	7
Documents	6
x-ray comparison	4
Video superimposition	5

The limitations of the methods used at the time were pointed out clearly, especially in the case of decomposed or skeletonized human remains. From both articles, one could sense that such human remains might only be identified by applying, at that point still insufficiently developed, methods of DNA identification, which was later proven to be correct.

## EARLY DNA IDENTIFICATIONS IN CROATIA: FROM A PROMISING NEW TOOL TO THE ROUTINE ANALYTICAL METHOD

**Figure 3:**
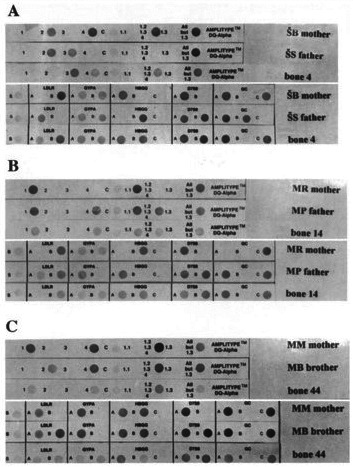
Results of the first (historical) DNA identification of skeletal remains found in Kupres mass graves by AmpliType^®^ PM+DQA1 PCR Amplification and Typing Kit (PerkinElmer Corp., Norwalk, CT, USA).

Unidentified skeletal remains from the Kupres area were selected for one of the very first DNA identifications of skeletal remains (Figure 3) from mass graves performed globally (3). These identifications were entirely performed in the local DNA facilities by the local scientists, with substantial help from their international collaborators. The importance of this effort was evidenced by several articles on this topic appearing in prominent scientific publications, including *Science* (18) and *JAMA* (19).

After these early successes achieved in collaboration with eminent American forensic scientists, new developments unfolded rapidly. Very early on, PCR-based techniques entered forensic genetics and found numerous applications, including war victim identification, disputed paternity testing, and crime scene evidence characterization. A turning point in human identification was the introduction of short tandem repeat (STR) markers, which shifted the power of exclusion in the testing of disputed paternity to a minimum of 99.999% and achieved high levels of discrimination in biological evidence discovered at crime scenes. It became evident that this novel multiplex system was substantially more informative in human identification than the early non-STR systems. In the analysis of more than 90 samples, the AmpliType^®^ PM + DQA1 systems successfully identified only 20%-25% of cases. The introduction of multiplex STR analysis and improvement of the original DNA isolation procedure increased the identification rate to 85% (20).

However, new challenges regarding statistical calculations arose. The main question was whether the set of only nine STR loci would be sufficient to attain a high degree of statistical confidence for identification purposes given the magnitude of human remains (21). Therefore, Croatian scientists began successfully employing more enhanced multiplex STR systems. Also, they soon realized that long bones produced extracts of higher quality compared with samples isolated from skulls or ribs (22).

Another important objective established around this time was to obtain relevant population data in Croatia and B&H to determine the frequency of allelic variants within the populations. The data are necessary to enable more accurate statistical calculations associated with genetic associations. Consequently, several studies addressed the genetic diversity of these populations, including the first one performed in collaboration with the FBI (23-29).

At the time, mitochondrial DNA (mtDNA) and Y-STRs were employed with different success rates. These markers were used in certain cases, depending on the context and the quantity and quality of the DNA. An innovative approach to simplifying this analysis was immobilized sequence-specific oligonucleotide (SSO) probe analysis of mtDNA hypervariable regions 1 and 2, again performed by a team of Croatian and US scientists (30). SSO probe analysis was used to determine the population variation of human mtDNA HV I and II in 105 Croatian individuals (31). The study reported successful SSO hybridization in 78% of the cases and a positive identification of one sample that was identical to a unique mtDNA sequence in a population of 105 randomly selected Croatians. This work confirmed that mtDNA analysis using immobilized SSO probes had its place in forensic DNA analysis of mass disaster remains of single and mass graves, particularly when the quality and quantity of remains did not yield full or nearly complete nuclear DNA profiles (30).

A limitation of this type of analysis is the lower power of discrimination due to the few common HVI/HVII sequences in European populations, a lower effective population size compared with autosomal markers, and a lack of recombination in the mtDNA genome. To address these problems, an improved linear array assay, mt HV+ HaploArray, was created. It targets additional polymorphic regions in both, non-coding and coding regions (32). The assay was validated for mtDNA analysis on bone samples, as they are often challenged forensic samples and the most common type of material in human remains. Additional population studies have contributed to confirming the improvement of the assay. The results showed a better distribution of mtDNA types (mitotypes) in the Croatian population (33,34). These studies showed that mtDNA typing can be a powerful identification tool in cases where nDNA analysis is not possible due to degradation or low copy.

In 2005, the Government of the Republic of Croatia officially published that, by the end of 1992, over 11 000 were reported missing in Croatia as a direct consequence of the war. This number corresponds with records indicating that 11 834 persons lost their lives. As a continuation to this report in 2005, experts who were included in the process from the very beginning published an article reviewing the identification efforts in Croatia after the war (13). Out of 3502 exhumed bodies, 2944 were identified and 558 remained unidentified. Overall, 1160 persons were still considered missing in 2005. (These numbers are updated with the more recent information presented at the beginning of this article.) The paper also listed the non-DNA and DNA-based methods of human identification that were used (Table 2). Furthermore, the authors emphasized that in war circumstances, with many victims lacking sufficient ante-mortem data and mostly buried in shared mass graves, identification becomes exceedingly complex and demanding. Traditional, non-DNA identification methods succeeded in identifying around 60% of the victims, while the identification of the remaining 40% required DNA analysis. Since non-DNA methods are getting less and less powerful as time passes, it was predicted that over the ensuing years, DNA analysis would become more important for victim identification (13).

**Table 2:** The success of DNA amplification for different bone types over a four-year period. Skeletal remains were from mass graves found after the war throughout Croatia and southern Bosnia and Herzegovina or given by Serbia and Montenegro. Source: Croat Med J. 2005;46:530–9, with permission (13)

Bone type	Number of DNA isolations in year						Percent of successfulness of DNA amplifications in year					
	2000	2001	2002	2003	2004	Total	2000	2001	2002	2003	2004	Total
Femur	15	69	46	76	13	219	80	91	87	97	100	92
Teeth	6	63	6	11	0	86	67	89	100	100	/	90
Skull	2	5	5	0	1	13	100	60	60	/	100	69
Humerus	1	6	6	0	3	16	100	67	100	/	67	81
Ulna	1	3	1	1	0	6	0	100	100	0	/	67
Radius	1	1	1	0	0	3	100	100	100	/	/	100
Mandibula	0	1	0	0	0	1	/	0	/	/	/	0
Rib	0	2	0	0	0	2	/	0	/	/	/	0
Calcaneus	0	0	1	0	0	1	/	/	0	/	/	0
Pelvis	0	0	4	0	0	4	/	/	75	/	/	75
Tibia	0	0	9	1	8	18	/	/	89	100	100	94
Sacrum	0	0	1	0	0	1	/	/	100	/	/	100
Fibula	0	0	2	0	0	2	/	/	100	/	/	100
Unknown	21	2	17	0	0	40	100	50	88	/	/	93
Total	47	152	99	89	25	412	87	86	87	97	96	89

## IDENTIFICATION OF WAR VICTIMS IN BOSNIA AND HERZEGOVINA: A MISSION WITHOUT PRECEDENT

Modern B&H is a multinational and multireligious country that has witnessed many conflicts throughout history, but at the same time, it is an interesting area regarding population genetics, history, and structure.

According to the Research and Documentation Center project from 2007, a total of 97 207 individuals lost their lives during the war in B&H from 1992 to 1995. These casualties comprised 64 036 Bosniaks, 24 905 Serbs, 7788 Croats, and 478 Others. Sarajevo during the four-year siege bore the brunt in terms of the number of victims, with 13 756 people killed, including 1601 children. It was followed by Srebrenica (8372 genocide victims), Prijedor, Zvornik, Bratunac, Foča, Vlasenica, Mostar, Doboj, Višegrad, Banja Luka, Brčko, and Rogatica.

Immediately following the end of the war, approximately 30 000 persons were estimated to be missing. According to the International Commission on Missing Persons (ICMP), the leading authority for this problem, 75% of these missing persons were accounted for, which is a ratio that has not been equaled in any other post-war country (35). Bosnian scientists, with the help of international colleagues and financial support from the international community, established one of the most efficient systems for DNA identification in the world. All these efforts were coordinated by the ICMP. Before ICMP established its own laboratories for DNA identification, it cooperated with the B&H institutions with DNA identification capacities, for example, the Institute for Genetic Engineering and Biotechnology (INGEB) from Sarajevo, the Clinical Center of the University of Tuzla, and individuals from Banja Luka. The ICMP’s efforts in the former Yugoslavia countries after the war resulted in an unparalleled accomplishment – the identification of over 27 000 (70%) of all missing persons (most cases being in B&H, where more than 30 000 persons were missing) (36).

**Figure 4:**
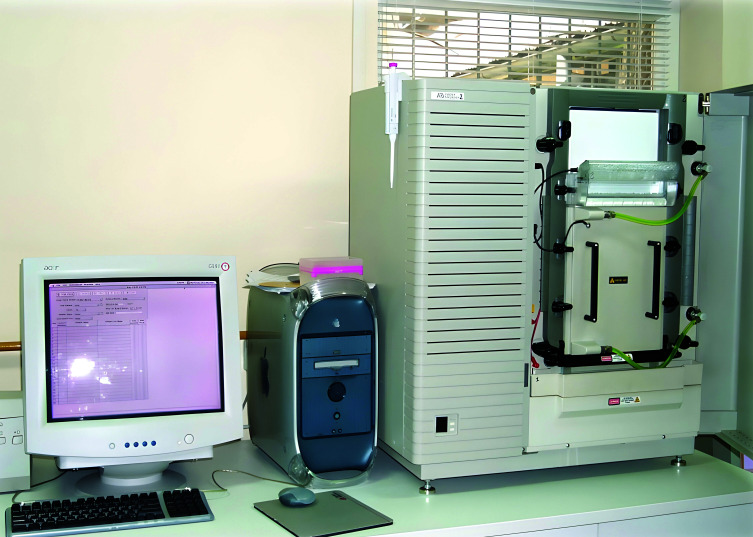
DNA laboratory INGEB-ICMP post-amplification room after reconstruction.

However, starting the process was challenging. Due to the destroyed infrastructure and many scientists from the field having left the country, it was necessary to create a completely new scientific team that would run the mission of identifying war victims. The first DNA laboratories were created in pre-existing buildings of scientific institutions. These spaces needed to be renovated to accommodate extremely restrictive laboratory conditions required for DNA analysis of skeletal remains (Figure 4). Additionally, in the late 1990s, DNA purification and analysis from skeletal remains was a challenge on its own. However, such difficulties motivated these scientists to innovate and optimize the existing protocols.

The first of these breakthrough papers was published in 2007, describing their original and highly effective DNA isolation protocol from the skeletal remains after years of optimization (37). This paper also overviewed the lab system for processing skeletal remains developed primarily at the INGEB, Sarajevo, and later, applied in a newly established ICMP laboratory. While this article presents the results of 20 femur samples, it is a summary of experience gained by processing thousands of skeletal remains in the years before the publication. Two other papers from the ICMP team presented unusual DNA typing results and analyzed the overall success rates of DNA typing (8,38).

## IDENTIFICATION OF THE OLD SKELETAL REMAINS: FROM THE SECOND WORLD WAR TO A MEMBER OF A MEDIEVAL ROYAL FAMILY

The same group of Slovenian, Croatian, and B&H scientists drew upon their experience with war victims in their effort to identify the remains from the Second World War (WWII) (14). The paper *DNA Identification of Skeletal Remains from the World War II Mass Graves Uncovered in Slovenia* reported one of the initial outcomes of human identification of old skeletal remains in this area (14). Considering the gap of 60 years between the body deposition and the time of excavation and analysis, this effort was a challenge due to low DNA quantity and quality and the presence of PCR inhibitors. However, the authors demonstrated that DNA identification was the only viable method of identification for these remains. Following a protocol optimization across all DNA typing steps, this study was one of the first identification studies of civilian WWII victims from a mass grave.

As an area with a turbulent history, the countries of the former Yugoslavia were inevitably impacted by WWII. Tens of thousands of people were reported missing during the war, as well as after it due to numerous mass executions carried out by the Yugoslav communists. With the establishment of democratic governments in these countries and following the requests from the missing persons’ relatives, efforts were made to identify the recovered remains from WWII. These efforts included the identification of mass grave victims in Škofja Loka, Slovenia (14).

**Figure 5:**
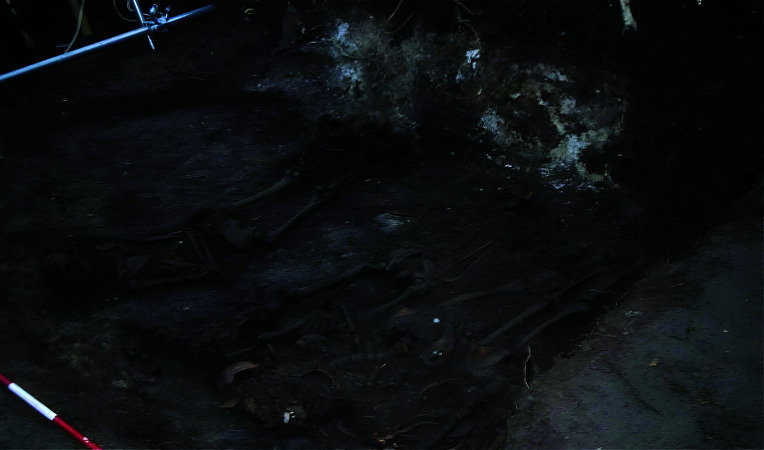
World War II mass grave Škofja Loka, Slovenia.

Two small mass graves were confirmed to contain the remains of 27 persons: 20 in the larger and seven in the smaller grave (Figure 5). According to the eyewitnesses’ testimony, the larger grave contained the bodies of Slovenian home guardsmen (German collaborators), while the smaller one contained the remains of seven German soldiers executed as war prisoners. The German soldiers, reportedly, buried the executed Slovenians and then excavated another grave for themselves. In total, 15 full and 12 partial DNA profiles were generated. A comparison of victims’ profiles against collected family reference samples resulted in four strong associations and subsequent positive identification of the remains. Moreover, five other profiles were possibly associated to the reference samples, though with a lower probability (14).

This study illustrated that the knowledge and proficiency acquired from the identification projects following the war in B&H and Croatia could effectively be applied to identify skeletal remains deposited in humid soil for a significantly longer time period. Fortunately, technologies available to forensic genetics are consistently being developed and enhanced so the power of DNA analysis as a human identification tool continues to improve.

Soon after, new DNA analysis tools, namely lineage markers Y-STRs and miniSTRs, became available for the analysis of WWII skeletal remains (39). Y-STR typing was used to identify two samples from Škofja Loka that were linked to family reference samples with insufficient probability of meeting an identification declaration (14). Y-STR analysis was used to assess if the two samples belonged to a paternal relative of two reference persons, since previously provided autosomal analysis presumed possible grandparent-grandson relationship. One case met the criterion for a positive match, but in the other, the paternal relation was not supported (39).

The identification of Škofja Loka mass grave victims also included a successful use of MiniSTR analysis. This type of analysis is especially useful in assaying markers in shorter amplicons that may recover information from degraded DNA samples. It was used to positively identify a woman who went missing in the autumn of 1942 based on the reference samples from her two sons (39). A summary of performed DNA analyses for the World War II skeletal remains from Slovenia is presented in Table 3. Taken together, these results demonstrated that both Y-STR and miniSTR analyses were valuable additional tools for human identification, especially in cases where autosomal STR analysis was insufficient.

**Table 3:** A summary of performed DNA analyses for the World War II skeletal remains from Slovenia. Source: Croat Med J. 2009;50:296–304, with permission (39)

Activity	Total number or percentage
Exhumed remains	48
Obtained profiles	41
Ratio of obtained profiles (%)	85.4
Reference samples	90
Positive identification	6
Ratio of positive identification (%)	12.5

**Figure 6:**
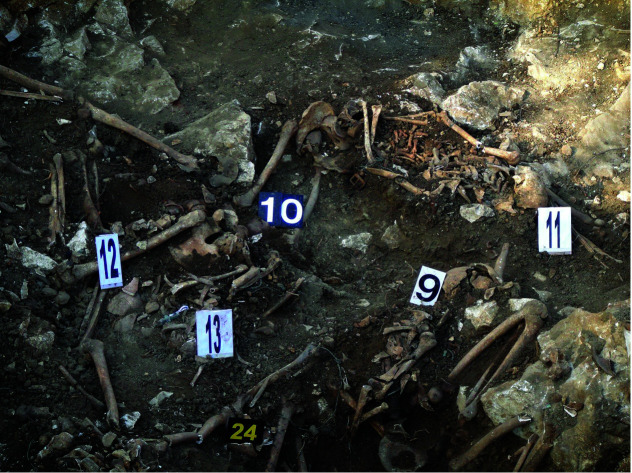
World War II mass grave Ljubuški, Bosnia and Herzegovina.

A few years later, almost the same team of scientists finalized the identification process of WWII skeletal remains from mass graves in Ljubuški, Herzegovina (40) (Figure 6). The success rate of DNA profiling of 10 persons was ~ 90%. Six positive identifications were made, all of them for skeletal remains of male individuals. These findings underscore the significance of a timely, targeted, and efficient sample collection from living relatives, as well as the deep emotional commitment of community members in identification of the remains.

**Figure 7:**
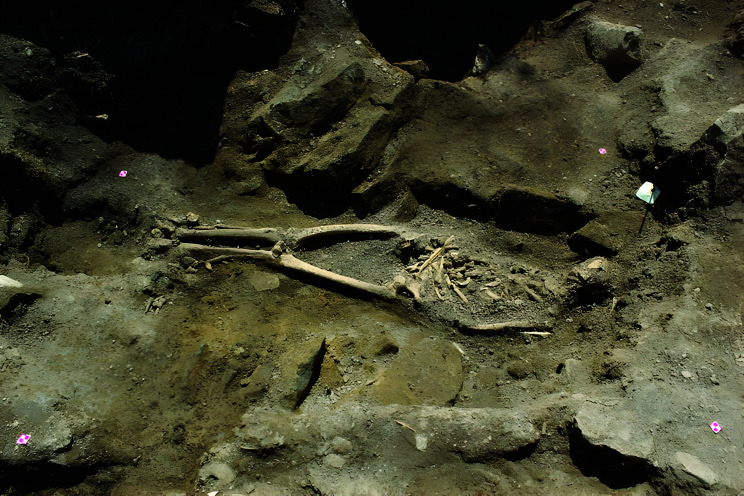
Skeletal remains from the 13th century (sample 27) in the grave of Zgošća, Bosnia and Herzegovina (41).

The techniques outlined above, or their minor modifications, were also used in the examination of archeological skeletal remains. Archeological material analysis is vital to understanding the human population’s history. Previous experience of local DNA experts and additional protocol optimizations were applied in the DNA analysis of bone fragments and teeth from ancient graves located in several archeological sites across B&H. The most important advantage of employing new multiplex STR systems was the capability to analyze minute quantities of degraded DNA and traces characterized by the existence of a high amount of PCR inhibitors. Archaeological cases, such as those of medieval remains from Zgošća (Figure 7) and Bobovac in B&H (41) and the case of Sister Marija Krucifiksa Kozulić in Croatia (42), showed the power of new STR analysis systems in the analysis of human remains several centuries old.

## CONCLUSIONS

This review article summarizes the expertise gained over the past three decades through identifying missing individuals in the former Yugoslav countries. These findings and experiences could be routinely applied without substantial alterations to analyzing skeletal remains from different eras and for current missing persons cases around the world. DNA analysis successfully identified remains from the 1990s, but also those retrieved almost seven decades earlier. Over the last three decades, genetic typing by STR analysis has emerged as the predominant method for human remains identification, thus evolving from an adjunct method to the choice forensic identification method, especially when other identification methods fail to provide viable lead data (43).

Nowadays, the adoption of enhanced or entirely novel methodologies increases the likelihood of successful nuclear DNA profiling of degraded skeletal remains, thereby augmenting the success rate of DNA profiling in such cases. Other methods, such as Y chromosome and mtDNA analysis and autosomal miniSTR systems, and particularly SNP analysis, allow the analysis even of the most challenging samples, such as those retrieved from archeological sites. Next-generation sequencing, high-volume SNP panels, and genealogical tools are likely the next tools that will increase the success of human identifications. Once again, forensic science helped to bring closure to families who had spent decades searching for their missing loved ones and enabled a dignified burial for the victims. As many of the closest relatives of the missing individuals are aging, it becomes urgent to collect samples from them and invest in technologies that improve the chances of typing success. Without their DNA profiles as the reference samples, the entire identification process would be significantly more complex, if not entirely unfeasible.
